# Tricyclic GyrB/ParE (TriBE) Inhibitors: A New Class of Broad-Spectrum Dual-Targeting Antibacterial Agents

**DOI:** 10.1371/journal.pone.0084409

**Published:** 2013-12-26

**Authors:** Leslie W. Tari, Xiaoming Li, Michael Trzoss, Daniel C. Bensen, Zhiyong Chen, Thanh Lam, Junhu Zhang, Suk Joong Lee, Grayson Hough, Doug Phillipson, Suzanne Akers-Rodriguez, Mark L. Cunningham, Bryan P. Kwan, Kirk J. Nelson, Amanda Castellano, Jeff B. Locke, Vickie Brown-Driver, Timothy M. Murphy, Voon S. Ong, Chris M. Pillar, Dean L. Shinabarger, Jay Nix, Felice C. Lightstone, Sergio E. Wong, Toan B. Nguyen, Karen J. Shaw, John Finn

**Affiliations:** 1 Trius Therapeutics, San Diego, California, United States of America; 2 ViviSource Laboratories, Waltham; 3 Micromyx LLC, Kalamazoo, Michigan, United States of America; 4 Advanced Light Source, Beamline 4.2.2, Berkeley, California, United States of America; 5 Lawrence Livermore National Laboratory, Physical and Life Sciences Directorate, Livermore, California, United States of America; Institut Pasteur, France

## Abstract

Increasing resistance to every major class of antibiotics and a dearth of novel classes of antibacterial agents in development pipelines has created a dwindling reservoir of treatment options for serious bacterial infections. The bacterial type IIA topoisomerases, DNA gyrase and topoisomerase IV, are validated antibacterial drug targets with multiple prospective drug binding sites, including the catalytic site targeted by the fluoroquinolone antibiotics. However, growing resistance to fluoroquinolones, frequently mediated by mutations in the drug-binding site, is increasingly limiting the utility of this antibiotic class, prompting the search for other inhibitor classes that target different sites on the topoisomerase complexes. The highly conserved ATP-binding subunits of DNA gyrase (GyrB) and topoisomerase IV (ParE) have long been recognized as excellent candidates for the development of dual-targeting antibacterial agents with broad-spectrum potential. However, to date, no natural product or small molecule inhibitors targeting these sites have succeeded in the clinic, and no inhibitors of these enzymes have yet been reported with broad-spectrum antibacterial activity encompassing the majority of Gram-negative pathogens. Using structure-based drug design (SBDD), we have created a novel dual-targeting pyrimidoindole inhibitor series with exquisite potency against GyrB and ParE enzymes from a broad range of clinically important pathogens. Inhibitors from this series demonstrate potent, broad-spectrum antibacterial activity against Gram-positive and Gram-negative pathogens of clinical importance, including fluoroquinolone resistant and multidrug resistant strains. Lead compounds have been discovered with clinical potential; they are well tolerated in animals, and efficacious in Gram-negative infection models.

## Introduction

Multidrug resistant (MDR) infections in the clinic are growing at a significant rate, largely due to the limited number of bacterial targets inhibited by the arsenal of antibiotics used for the last half-century [1-3]. Since the 1960s, the carbapenems (a β–lactam natural product antibiotic class introduced in the 1980s) and the fluoroquinolones are the only new classes of antibiotics that have been developed with activity against clinically important Gram-negative pathogens. The difficulty in developing new antibacterial classes stems from the challenges of developing small molecules capable of penetrating the cell envelope and avoiding drug efflux systems [3]. As a result, there is an alarming lack of efficacious therapeutic choices for clinicians treating these infections. To provide potential solutions to this problem, we used structure-based drug design (SBDD) to develop a novel class of broad-spectrum antibacterial agents with activity against resistant pathogens, including Gram-negative MDR strains.

Advances in SBDD technology combined with a greater understanding of the factors that influence Gram-negative permeability and drug efflux has made possible the rational design of broad-spectrum antibacterial agents. Target selection is central to this process. Targets need to meet key criteria: First, the active-site of the target needs characteristics that allow for the design of highly potent enzyme inhibitors (subnanomolar *K*_i_). In our experience, this level of enzymatic activity is required to generate sufficient antibacterial potency on Gram-negative organisms, particularly for cytoplasmic targets. Next, the inhibitor target needs to be both unique to and conserved among bacteria, to enable the development of inhibitors that are both selective for bacteria and have a broad antibacterial spectrum. Further, the inhibitor-binding site needs to be distinct from the sites targeted by existing drugs to avoid cross-resistance with established antibiotic classes. Additionally, finding conserved target pairs from different essential pathways that could be inhibited by a single agent is desirable, as dual-targeting agents raise the statistical barrier to the development of target-based resistance that plagues many single-targeting agents [2]. Finally, the active-site of the target needs to be compatible with inhibitors possessing features necessary for Gram-negative penetration and retention, namely, low molecular weight, sufficient hydrophilic character and functional groups with ionizable centers at physiological pH.

The ATP binding subunits of the bacterial topoisomerases DNA gyrase (GyrB) and topoisomerase IV (ParE) meet the criteria described above. Both enzymes modify the topological state of DNA in an ATPase-dependent manner to allow replication: DNA gyrase is primarily responsible for the initiation of DNA replication and elongation of nascent DNA, while topoisomerase IV is primarily responsible for decatenation of daughter chromosomal DNA at the end of replication [4]. These topoisomerase complexes are validated drug targets. DNA gyrase (GyrA/GyrB) and topoisomerase IV (ParC/ParE) are the targets of the fluoroquinolones but these agents bind at the interfaces between the GyrA and ParC subunits and the GyrB and ParE subunits, respectively [5]. Growing resistance to fluoroquinolones, frequently mediated by mutations in the drug-binding site, is increasingly limiting the utility of this antibiotic class [6], prompting the search for other inhibitor classes that target different sites on the topoisomerase complexes. This has led to substantial activity by many groups focused on the development of inhibitors targeting the ATPase sites on GyrB and ParE [7]. The natural product novobiocin (discovered in the 1950s) has been shown to kill Gram-positive bacteria *via* inhibition of GyrB, but failed in the clinic due to problems with toxicity [8]. In addition to issues with safety, the size, large binding contact surface and lack of dual-targeting activity (i.e. weak activity against ParE) results in the rapid development of resistance to novobiocin [5]. A number of other discovery programs aimed at the development of superior GyrB/ParE targeting antibacterial agents have provided support for the concept that SBDD could yield more potent GyrB or GyrB/ParE inhibitors [7]. However, none have been successful in generating an inhibitor series with broad-spectrum antibacterial activity or advancing a molecule into the clinic. The success of GyrB/ParE inhibitor discovery programs has been hampered by difficulties in creating inhibitors with balanced dual-targeting activity [9], and, more universally, by difficulties in developing inhibitors with the necessary enzymatic potencies and physicochemical property profiles to elude multi-drug efflux pumps in most Gram-negative pathogens [10-12]. Problems with high serum-protein binding have also been noted [10,13], potentially compromising the effectiveness of inhibitors to kill bacteria *in vivo*. We focused on the highly conserved regions of the two targets, particularly regions with polar character, to design compounds with both enzymatic potency and physicochemical properties profiles needed for Gram-negative antibacterial activity. We have succeeded in creating a novel **Tri**cyclic class of Gyr**B**/Par**E** dual-targeting pyrimidoindole inhibitors (TriBE inhibitors) with potent, broad-spectrum antibacterial activity against a wide range of bacterial pathogens that include drug resistant strains of *Pseudomonas aeruginosa*, *Acinetobacter baumannii* and *Klebsiella pneumoniae*. Several lead compounds have been generated, including representative inhibitors described herein.

## Results and Discussion

### Structure Guided Discovery and Optimization of the Pyrimidoindole Inhibitor Scaffold

**Figure 1:**
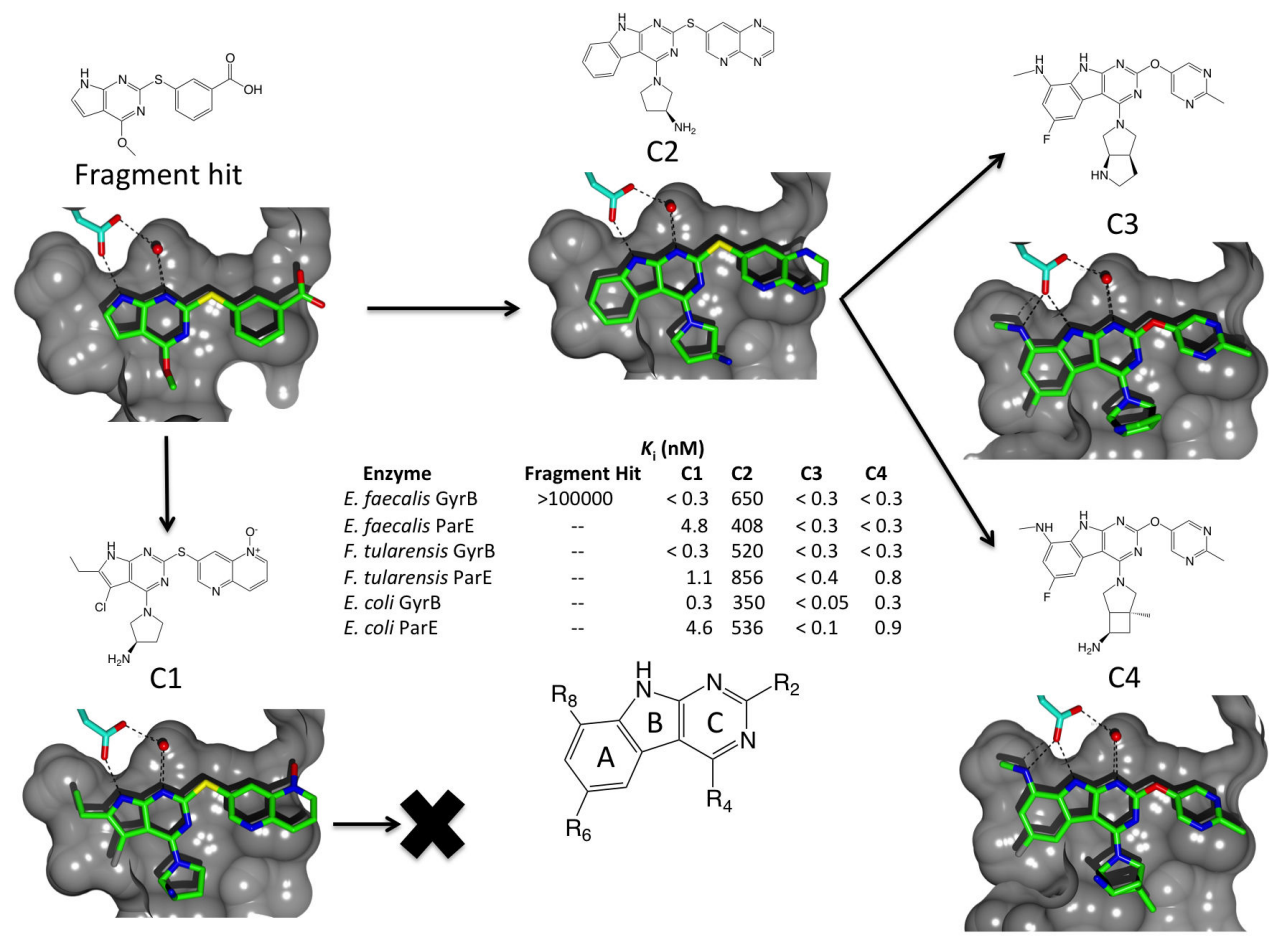
Optimization of inhibitor scaffolds. For the fragment hit and inhibitor candidates **C1**, **C2**, **C3** and **C4,** identical cutaway views of solvent accessible surface representations of the active-site pockets of *E. faecalis* GyrB from the crystal structures of complexes of the inhibitors with the 24 kDa N-terminal fragment of GyrB from *E. faecalis* GyrB are shown. The bound inhibitors are drawn with green bonds, the conserved ATP-binding aspartate is drawn with blue bonds and the structural water molecule that plays a key role in substrate binding in GyrB and ParE is shown as a red sphere. Potential hydrogen-bonds between the inhibitors, aspartate and water molecule are depicted as dotted lines. Optimization of the pyrrolopyrimidine scaffold led to inhibitors like **C1** with good enzyme potency but only moderate Gram-negative antibacterial activity [16]. Expansion of the bicyclic pyrrolopyrimidine scaffold to a tricyclic pyrimidoindole scaffold **(C2)** fills an interior lipophilic pocket and offers superior optimization vectors to improve enzyme potency. Subsequent elaboration of the tricyclic scaffold with a fluorine atom at R_6_ and an aminomethyl moiety at R_8_ dramatically improved inhibitor potency and ligand efficiency. The 6-fluoro-*N-*methyl-9*H*-pyrimido[4,5-*b*]indol-8-amine scaffold quantitatively fills the lipophilic interior sub-pockets of the GyrB/ParE active-sites and adds a new hydrogen-bond. **C3** and **C4** demonstrate sub-nanomolar enzyme potency versus GyrB and ParE enzymes from a broad range of Gram-positive and Gram-negative pathogens; inhibition constants (*K*_i_ values) are shown for a representative enzyme panel that includes the full length GyrB and ParE enzymes from *E. faecalis*, Francisella tularensis, and *E. coli*.

Crystallographic fragment screening was employed to identify starting scaffolds. Similar to the “needle” screen conducted by scientists at Roche [14], low molecular weight fragments with adjacent hydrogen-bond donor/acceptor moieties that engage the ATP adenine-binding aspartate and structural water in the active-site pocket were selected for screening [15]. Based on an analysis of the binding modes of fragment hits on *Enterococcus faecalis* GyrB, a pyrrolopyrimidine scaffold was deemed an appealing candidate for optimization because it projected synthetic vectors towards all the highly conserved sub-pockets of the GyrB and ParE active-sites including a site for the introduction of charged functionality [15] (Figure 1).

**Figure 2:**
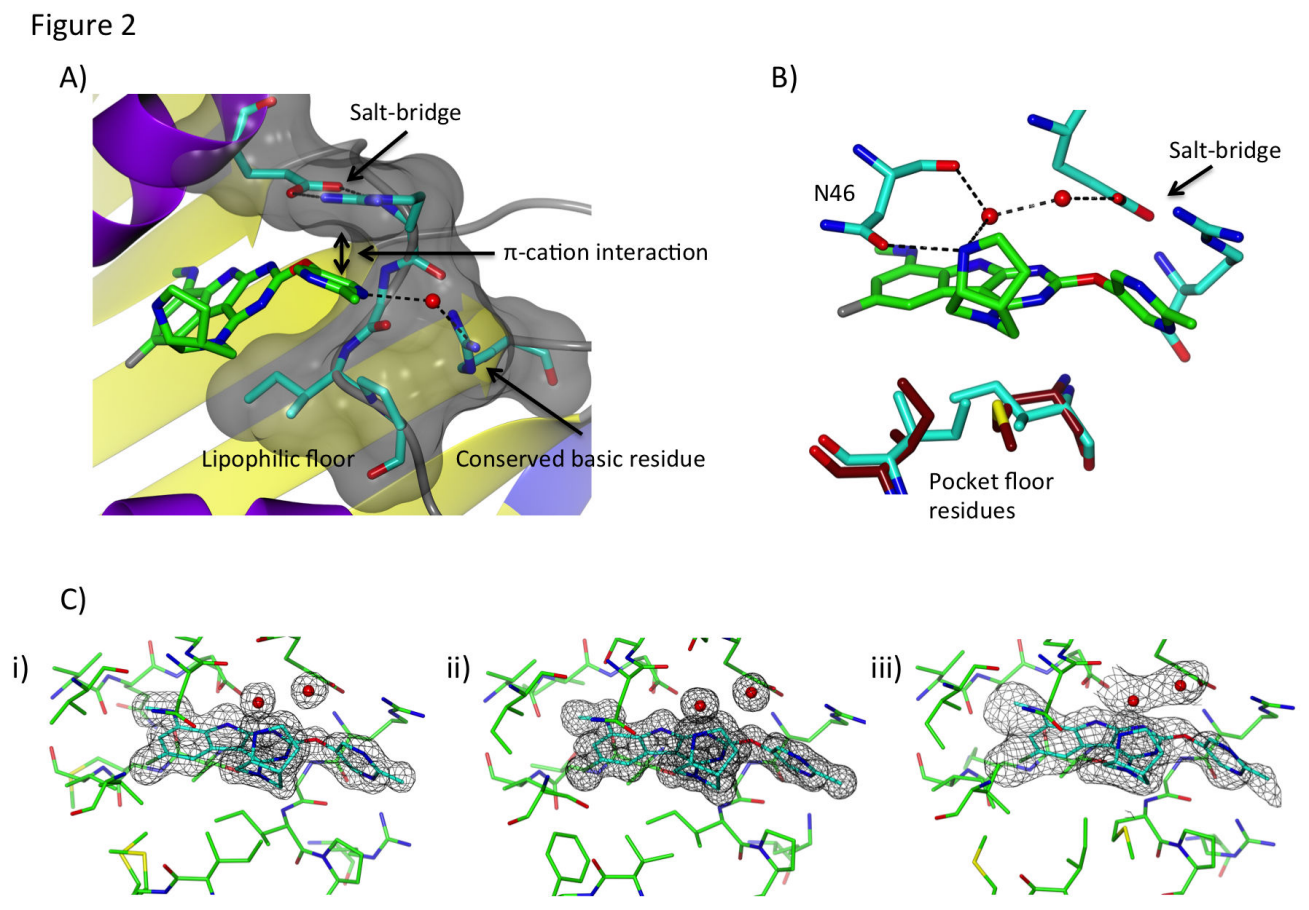
Summary of inhibitor optimization strategies. **A**) Side view of the “salt-bridge” pocket from the crystal structure of the complex of *E. coli* GyrB with **C3**, with key interactions highlighted. **C3** is drawn with green bonds. Potential hydrogen-bonds are depicted as dotted lines. The residues comprising the salt-bridge pocket are drawn with blue bonds and a semi-transparent surface representation of the pocket is shown. The salt-bridge pocket residues curl around the R_2_ pyrimidine, forming a U-shaped pocket. R_2_ substituents were designed to address the complex structural and electronic features of the salt-bridge pocket. Extensive *ab* *initio* and binding free energy calculations of the R_2_ methyl pyrimidine of **C3** show significant binding energy from a π–cation interaction with the salt-bridge Arg. The methyl pyrimidine also engages the Arg on the outer rim of the salt-bridge pocket through a water-mediated hydrogen-bond. Van der Waals interactions are observed between a conserved proline that defines the face of the salt-bridge pocket opposite the Glu-Arg salt-bridge pair. **B**) Alternate view of the *E. coli* GyrB complex structure with **C3**, highlighting key polar interactions between the R_4_ diamine of **C3**, active-site residues and an ordered solvent network. The Asn residue shown in the figure (N46 from the *E*. *coli* structure) and salt-bridge residues are conserved in all bacterial topoisomerases, while the residues comprising the “pocket floor” (blue for the residues in *E. coli* GyrB, tan for the residues from the overlaid *F. tularensis* ParE/C3 complex structure differ between GyrB and ParE enzymes. The R_4_ diamine sits at the protein-solvent interface at the outer rim of the lipophilic interior pocket that binds the (A) ring of the inhibitor. The upper face of the inhibitor occupies a highly conserved polar pocket while the lower face occludes a lipophilic shelf (the pocket floor) that is structurally heterogeneous between GyrB and ParE due to sequence differences in the enzymes, as highlighted. The R_4_ diamine adopts a low energy conformation that does not impinge on the structurally diverse pocket floor and directs a basic amine out of the pyrimidoindole plane to interact *via* hydrogen-bonds with the conserved Asn at the mouth of the interior pocket (N46). The basic amine complements the negative electrostatic potential in this region of the active-site; the same anionic pocket captures the terminal amine from a conserved lysine residue involved in phosphate binding in the dimeric complex of *E. coli* ParE with ADPNP [17]. The R_4_ diamine also hydrogen-bonds with an ordered solvent network above the pocket floor. The water molecules from this network shown in the figure were observed in similar positions in GyrB and ParE crystal structures from multiple orthologs (as shown in **C**), and molecular dynamics simulations showed this water network remained throughout all simulations, and each water molecule had significantly long residence times. Thus, these water molecules were treated as conserved structural elements during inhibitor optimization. **C**) Electron density maps showing the positions of **C3** and the water network that was conserved across all GyrB and ParE enzymes that were structurally characterized in this study. Final 2|fo-fc| electron density maps contoured at 1.3σ for: **i**) the 1.6 Å *E. coli* GyrB complex with **C3**, ii) the 1.3 Å *E. faecalis* GyrB complex with **C3**, and iii) the 2.4 Å *F. tularensis* ParE complex with **C3**. The water network that interacts with the R_4_ diamine of the inhibitor is conserved in the three protein orthologs. R_4_ groups were designed to position an amine that simultaneously hydrogen-bonds with the water network and a conserved Asn residue in the active-site pocket.

**Figure 3:**
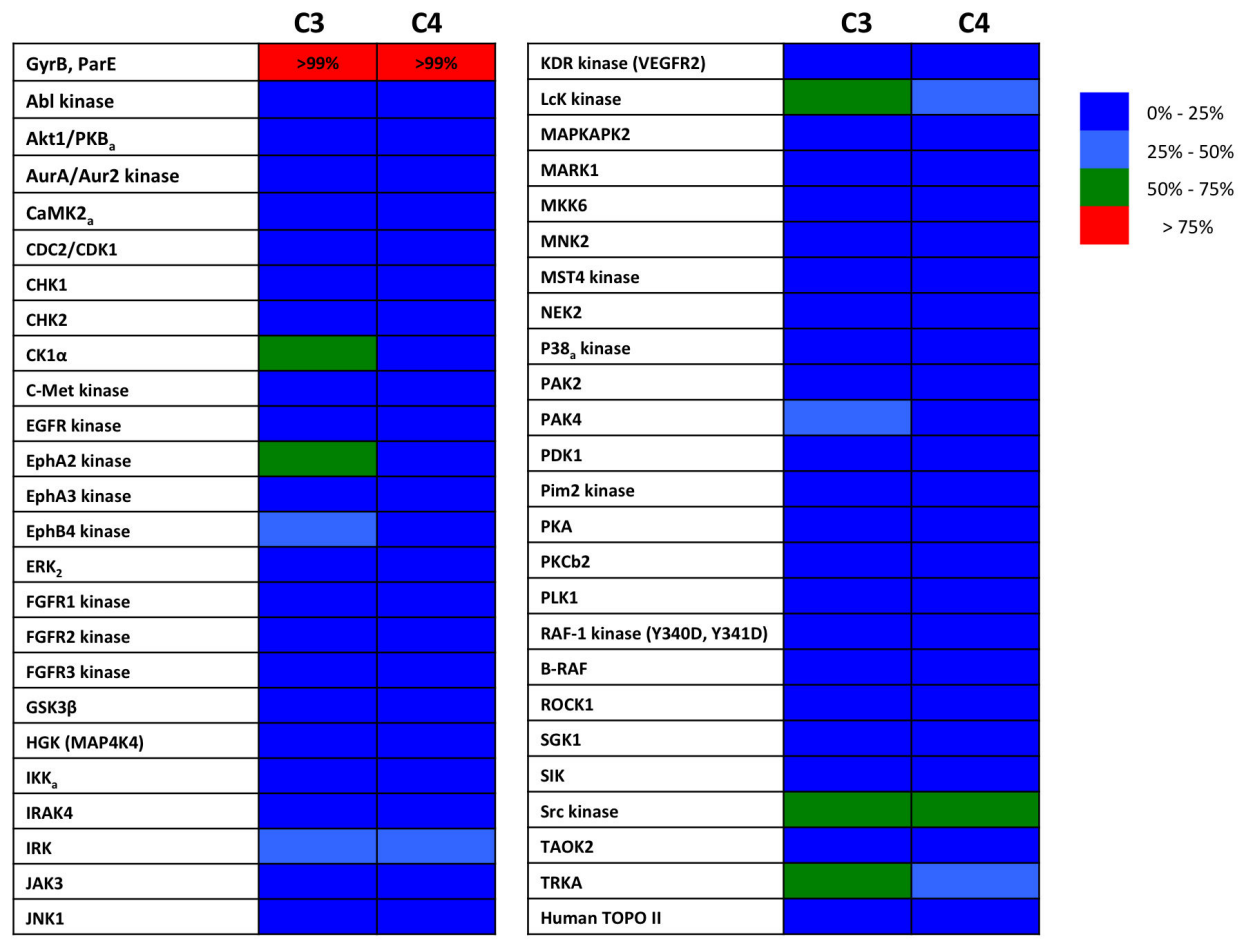
Selectivity of TriBE inhibitors versus eukaryotic ATP-binding proteins. Inhibitory activities of **C3** and **C4** against a panel of divergent human kinases, and human topoisomerase II. All compounds were assayed at 10 μM concentration. Level of inhibition is color-coded as indicated in the inset.

Initial SBDD efforts were successful in converting the fragment hit into a pyrrolopyrimidine inhibitor series (e.g. **C1**) that had both the desired broad enzymatic spectrum and dual-targeting enzymatic profile by expanding the scaffold into the lipophilic interior of the GyrB and ParE active-sites [15,16] (Figure 1). However, activity against Gram-negative pathogens was limited and we ultimately exhausted avenues for improving potency at the enzyme level without elevating compound molecular weight and lipophilicity to a degree that further compromised Gram-negative antibacterial activity and spectrum. Consequently, we examined multiple alternative inhibitor scaffolds that could exploit the structure-activity relationships (SAR) from this initial series while providing greater potency and antibacterial spectrum. Dramatic improvements in inhibitor potency, properties and antibacterial activity were achieved by switching to a pyrimidoindole scaffold (**C2**, Figure 1). The phenyl (A) ring of the pyrimidoindole scaffold filled the interior pocket and presented superior optimization vectors that allowed for the design of inhibitors that structurally and electronically complement the active-site. An aminomethyl substituent was added to engage the conserved adenine-binding Asp residue with an additional hydrogen-bond while occupying a recessed lipophilic pocket. Shape and charge complementarity to the conserved regions of both GyrB and ParE was further improved by adding a fluorine substituent at the R_6_ position. Analysis of representative GyrB and ParE structures from multiple bacterial species revealed subtle differences in the shapes and volumes of the active-site pockets in the vicinity of the R_6_ substituent. A fluorine substituent at R_6_ was accommodated well in all enzymes, and was optimal for broad enzymatic spectrum and dual-targeting (Figure S1). The binding mode of the pyrimidoindole scaffold oriented the R_2_ and R_4_ vectors on the (C) ring in directions that were exploited to incorporate substituents that interact with two highly conserved binding pockets at the receptor solvent interface in GyrB and ParE (Figure 2). As a result, we were able to create a highly potent, ligand efficient tricyclic inhibitor series (e.g. **C3** and **C4**) with broad enzymatic spectrum. Since GyrB and ParE are structurally unique and belong to a class of ATP-binding proteins called the GHKL superfamily of ATPases/kinases with an unconventional fold [18], the potent TriBE lead compounds are highly selective for their bacterial targets when compared to eukaryotic ATP-binding proteins like protein kinases and human topoisomerase II (Figure 3). This suggests that TriBE inhibitors will not suffer from those potential sources of off-target toxicity in the host. 

### Optimization of Antibacterial Activity and Spectrum

Parallel optimization of enzyme inhibitor potency and antibacterial activity revealed that modulation of the charge state of the pyrimidoindole inhibitors strongly influenced antimicrobial activity, particularly against Gram-negative organisms. Diamine substituents at R_4_ like the (3aR,6aR)-octahydropyrrolo[3,4-*b*]pyrrole used in **C3** were observed to significantly enhance Gram-negative antibacterial activity and spectrum by improving both enzyme inhibitor potency and imparting physicochemical characteristics to inhibitors that addressed the unique characteristics of the Gram-negative cell envelope. Drug efflux pumps and the orthogonal sieving properties of the inner and outer membranes of the Gram-negative cell envelope pose significant barriers to the development of antibacterial agents that need access to cytoplasmic targets; the outer membrane is selectively permeable to small charged or polar molecules *via* porins, while the inner membrane lipid bilayer is essentially impermeable to charged ions, but contains efflux pumps that actively transport lipophilic molecules out of the cell [19,20]. Fluoroquinolones and tetracyclines, which are active against Gram-negative bacteria, equilibrate between neutral (minor) and charged (predominant) states at physiological pH [20]. The charged species traverse the outer membrane and are poor efflux pump substrates, while the neutral species are able to diffuse across the inner membrane lipid bilayer [20]. The ionizable R_4_ diamines used in our pyrimidoindole inhibitors are similar to the diamine substituents used in many fluoroquinolone antibiotics [21]. The relative distributions of charged/neutral species in **C3** and **C4**, calculated using the pKa calculation algorithm [22] in the Advanced Chemistry Development, Inc. software package, are estimated to be in the 95:5 to 90:10 range at pH 7.4. These ratios of charged to neutral species at physiological pH are similar to those calculated for norfloxacin, amifloxacin and perfloxacin [20], and are likely responsible for enabling efficient penetration of the Gram-negative cell envelope.

**Figure 4:**
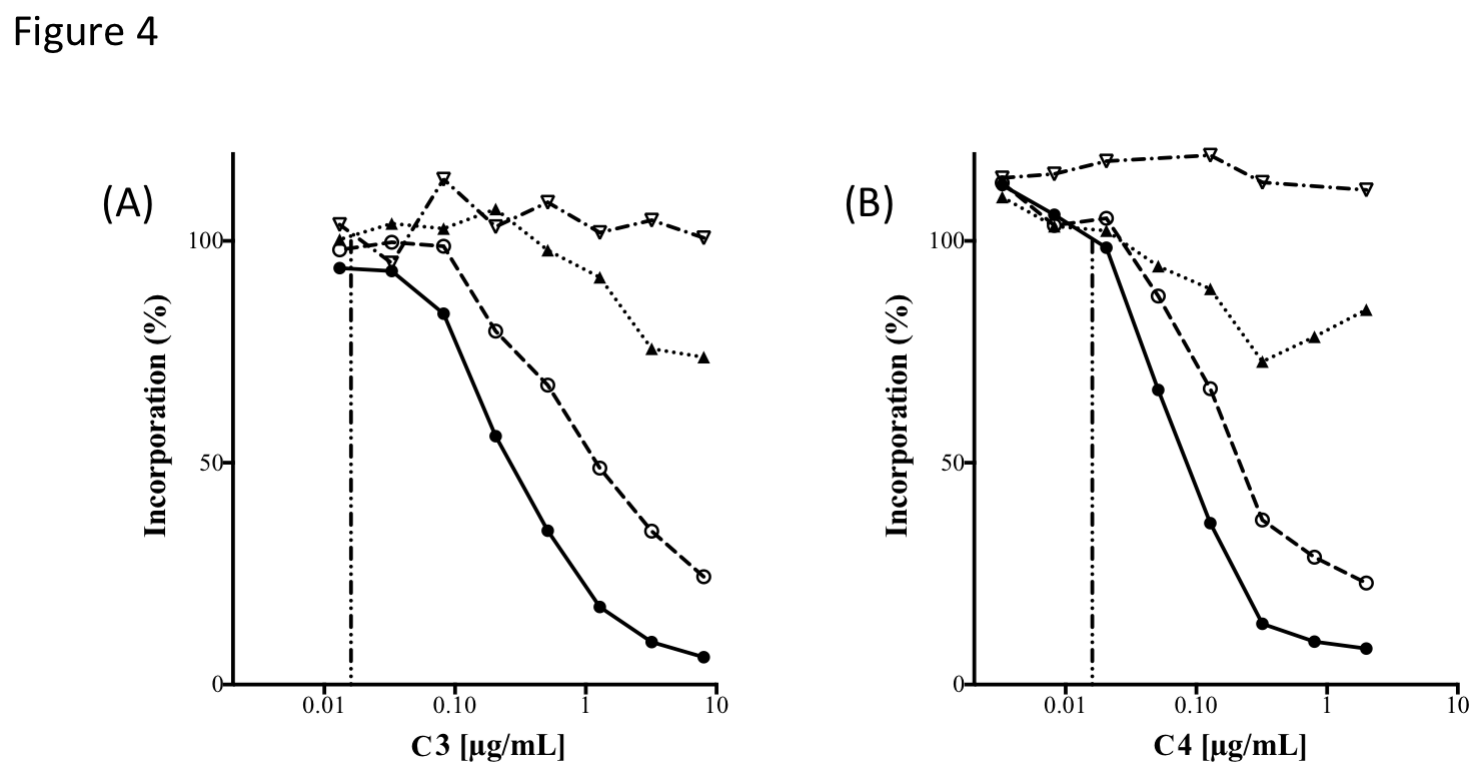
The effects for C3 (A) and C4 (B) on macromolecular synthesis in *E. coli* (BAS849) *imp*. Incorporation of [^3^H]-precursors of DNA (●), RNA (○), protein (▲) and cell wall (▽) was examined. The MIC value for each compound is indicated by a vertical dashed line. Both compounds exert a primary effect on DNA synthesis and a secondary effect on RNA synthesis.

The *in vitro* antibacterial activities of **C3** and **C4** have been evaluated against a diverse array of clinical pathogens, including those with important resistance phenotypes. A summary of the results using representative pathogens is shown in Tables 1 and 2. As expected, cross-resistance with existing antibiotic drug classes was not observed with any resistance phenotype tested. To confirm that the pyrimidoindole lead compounds kill bacterial cells *via* inhibition of GyrB and ParE, macromolecular synthesis inhibition assays were conducted (Figure 4), and demonstrated that **C3** and **C4** specifically inhibit DNA (primary) and RNA (secondary) synthesis. This pattern of macromolecular synthesis inhibition has been observed for other validated GyrB/ParE inhibitors including novobiocin [16,23]. 

**Table 1:** MIC ranges and MIC_90_ values of C3, C4 and selected antibiotic controls for important clinical Gram-positive pathogens.

**Organism (number of clinical isolates tested)**	**Antimicrobial Agent^*a*^**	**MIC (μg/mL)**	
		**Range**	**MIC_90_**
*Staphylococcus aureus*	**C3**	**0.008 - 0.06**	**0.06**
(17)**^*b*^**	**C4**	**0.002 - 0.008**	**0.008**
	CIP	0.5 - > 4	> 4
	LZD	2 - > 16	4
	VAN	0.25 - 4	1
*Streptococcus pneumoniae*	**C3**	**≤ 0.001 - ≤ 0.001**	**≤ 0.001**
(17)**^*c*^**	**C4**	**≤ 0.001 - ≤ 0.001**	**≤ 0.001**
	CIP	1 - > 4	> 4
	LZD	0.5 - 2	2
	VAN	0.25 – 0.25	0.25
*Enterococcus faecalis*	**C3**	**≤ 0.001 – 0.002**	**≤ 0.001**
(10)**^*d*^**	**C4**	**≤ 0.001 - ≤ 0.001**	**≤ 0.001**
	CIP	0.5 - > 4	> 4
	LZD	1 -16	4
	VAN	0.5 - > 16	> 16

^a^ CIP = ciprofloxacin, LZD = linezolid, VAN = vancomycin

^b^ All strains are methicillin-resistant (MRSA)

^c^ Includes penicillin and fluoroquinolone-resistant strains

^d^ Includes vancomycin (VRE) and fluoroquinolone-resistant strains

**Table 2:** MIC ranges and MIC_90_ values of C3, C4 and selected antibiotic controls for important clinical Gram-negative pathogens.

**Organism (number of clinical isolates tested)**	**Antimicrobial Agent** ^a^	**MIC (μg/mL)**	
		Range	MIC_90_
*Haemophilus influenzae*	**C3**	**0.015 - 0.25**	**0.12**
(11)**^*b*^**	**C4**	**0.03 - 0.5**	**0.25**
	AMP	0.12 - 2	2
	AZM	1 - 8	4
	CFZ	0.5 - 8	8
*Moraxella catarrhalis*	**C3**	**≤ 0.008 - ≤ 0.008**	**≤ 0.008**
(10)	**C4**	**≤ 0.008 - ≤ 0.008**	**≤ 0.008**
	AMP	≤ 0.015 - 16	16
	AZM	≤ 0.015 - 0.5	0.25
	CFZ	0.5 - 4	2
*Escherichia coli*	**C3**	**0.12 - 1**	**1**
(22)**^*c*^**	**C4**	**0.06 - 0.5**	**0.5**
	CIP	0.008 - > 128	> 128
	GEN	0.25 - > 64	64
	IMP	0.06 - 1	0.25
*Klebsiella pneumoniae*	**C3**	**0.25 - 8**	**2**
(31)**^*d*^**	**C4**	**0.25 - 4**	**1**
	CIP	0.015 - > 128	128
	GEN	0.12 - > 64	> 64
	IMP	0.12 - > 64	32
*Acinetobacter baumannii*	**C3**	**0.03 – 0.5**	**0.5**
(20)**^*e*^**	**C4**	**0.03 – 0.25**	**0.25**
	CIP	0.12 - > 128	> 128
	GEN	≤ 0.06 - > 64	> 64
	IMP	0.12 - 64	64
*Pseudomonas aeruginosa*	**C3**	**1 - 8**	**8**
(15)**^*e*^**	**C4**	**0.25 - 2**	**2**
	CIP	0.06 - 64	32
	GEN	0.5 - 64	16
	IMP	0.5 - > 64	64

^a^ CIP = ciprofloxacin, AMP = ampicillin, AZM = azithromycin, CFZ = cefuroxime, GEN = gentamicin and IMP = imipenem.

^b^ Includes β-lactamase negative, ampicillin-resistant strains (BLNAR)

^c^ Includes extended spectrum β-lactamase positive (ESBL) and fluoroquinolone-resistant strains

^d^ Includes extended spectrum β-lactamase positive (ESBL), *K. pneumoniae* carbapenemase positive (KPC), New Delhi metallo-β-lactamase positive (NDM-1) and fluoroquinolone-resistant strains

^e^ Includes imipenem and fluoroquinolone-resistant strains

Both inhibitors were exceptionally potent against all Gram-positive and fastidious Gram-negative pathogens screened, with MIC_90_ values below 0.1 μg/mL in nearly all cases. **C3** and **C4** retained good antimicrobial activity against clinically problematic Gram-negative pathogens, including the highly drug resistant *K. pneumoniae* carbapenemase positive (KPC) and New Delhi metallo-β-lactamase positive (NDM-1) strains (Table 3). The dual-targeting activities of **C3** and **C4** effectively suppressed emergence of resistance; both compounds demonstrate spontaneous resistance frequencies in *E. coli* below 2 x 10^-11^ at concentrations 4-fold above the MIC (Table 4). The moderately elevated MIC values observed for some Gram-negative pathogens are likely due to efflux pump activity; this hypothesis is consistent with differences in MIC values observed between isogenic wild-type and pump knockout strains of *Escherichia coli* (Table 3). The MIC values were not affected by the addition of 20% mouse serum (Table 3), improving the prospects for the effectiveness of this inhibitor in *in vivo* infection models. 

**Table 3:** MIC values for selected strains.

	**MIC (μg/mL)**		**MIC (μg/mL) in 20% mouse serum**	
**Bacterial Strain**	**GP-3**	**GP-4**	**GP-3**	**GP-4**
*Staphylococcus aureus* (ATCC 13709)	0.008	0.004	0.004	0.004
*Streptococcus pneumoniae* (ATCC 51916)	0.001	0.001	--	--
*Enterococcus faecalis* (ATCC 29212)	0.002	0.002	--	--
*Haemophilus influenzae* (ATCC 49247)	0.031	0.031	--	--
*Escherichia coli* (ATCC 25922)	0.125	0.125	0.06	0.125
*Escherichia coli* (MCR106) (parent of BAS849)	1	0.5	--	--
*Escherichia coli* (BAS849) *imp*	0.008	0.004	--	--
*Escherichia coli* (BW25113)	1	1	--	--
*Escherichia coli* (BW25113*ΔtolC*)	0.016	0.008	--	--
*Acinetobacter baumannii* (ATCC 19606)	0.125	0.25	--	--
*Klebsiella pneumoniae* (ATCC 700603)	2	1	--	--
*Klebsiella pneumoniae* NDM-1 (ATCC BAA2146)	2	1	--	--
*Klebsiella pneumoniae* KPC-1 (ATCC BAA1705)	2	1	--	--
*Pseudomonas aeruginosa* (PAO1)	2	1	--	--

**Table 4:** Spontaneous incidence of resistance in *E. coli* (ATCC 25922).

**Selecting Agent**	**Mutation Frequency (at 4X MIC)**
Ciprofloxacin	3.3 x 10^-9^
**C3**	< 1.9 x 10^-11^
**C4**	< 1.9 x 10^-11^

MIC values:

Ciprofloxacin 0.008 μg/mL

**C3** 0.25 μg/mL

**C4** 0.125 μg/mL

### *In Vivo* Efficacy

**Figure 5:**
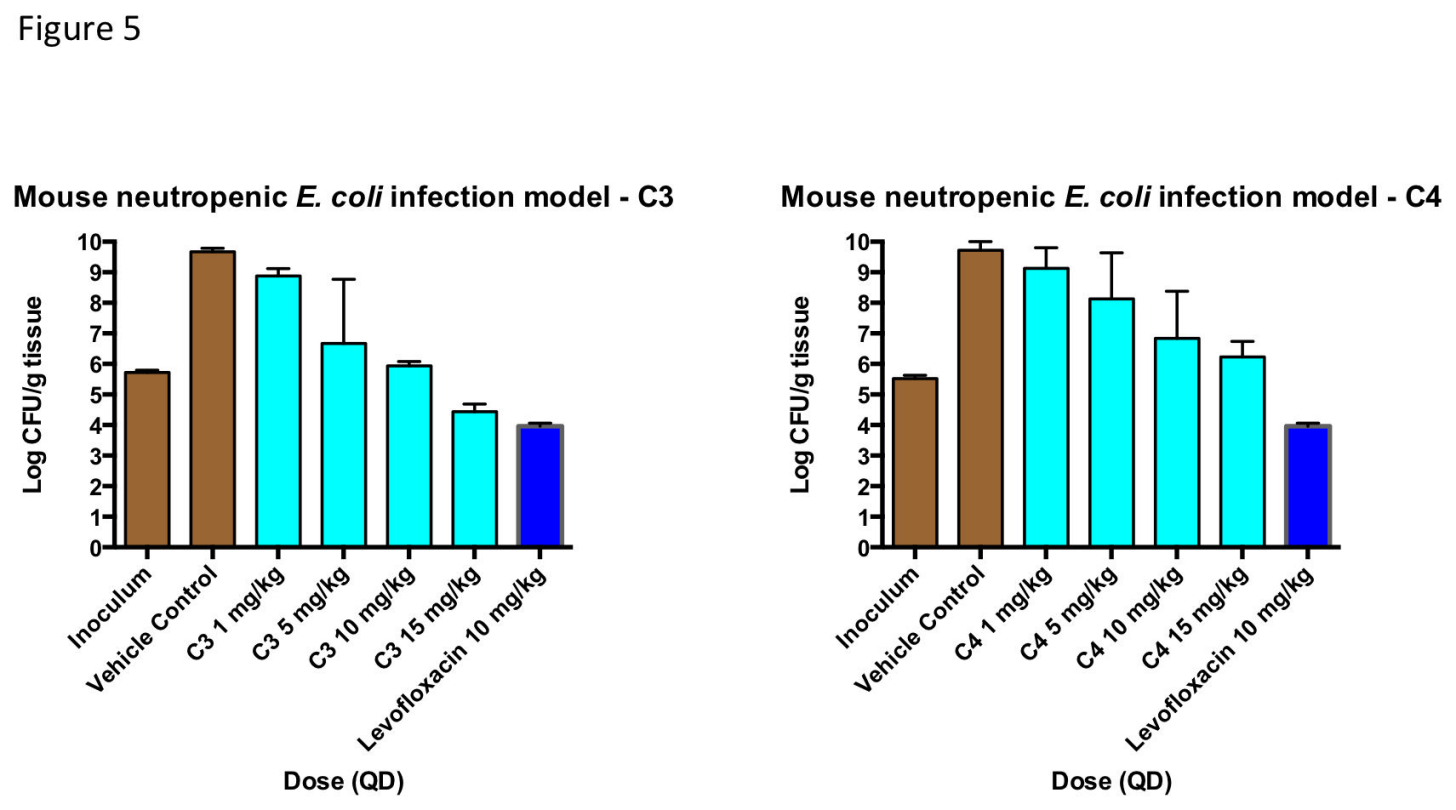
Reduction in bioburden after 24 hrs in a neutropenic mouse thigh infection model. The MIC values for **C3**, **C4** and levofloxacin against the *E. coli* strain used in the study are 0.13, 0.13 and 0.03 μg/mL, respectively.

The *in vitro* antimicrobial activities of **C3** and **C4** translated well in *in vivo* infection models. Both compounds demonstrated efficacy in several intravenous Gram-negative mouse infection models, including an *E. coli* neutropenic mouse tissue infection model (Figure 5). Single doses of 5-15 mg/kg reduced the tissue bioburden in mouse thigh by 3 log_10_ CFU within 24 hours, as compared with a vehicle control. Notably, a single 15 mg/kg dose of **C3** reduced tissue bioburden by > 1 log below the stasis level, while the same dose of **C4** reduced bioburden to approximately the stasis level.

### Conclusions

The failure by industry and academia to deliver new antibiotic classes with Gram-negative spectrum over the last several decades highlights the difficulty of the problem. The technical challenges to realizing this goal are becoming addressable due to advances in SBDD combined with a growing understanding of the sieving properties of the Gram-negative cell envelope. The discovery of the TriBE inhibitors demonstrates an approach with general applicability. The selection of suitable targets combined with an optimization strategy that placed equal emphasis on enzymatic potency, spectrum and drug-properties has led to the discovery of an exciting new class of broad-spectrum antibacterial agents with potent activity against dangerous multi-drug resistant Gram-negative pathogens. The TriBE inhibitor series is undergoing further optimization and advancement to preclinical evaluation for utilization in the clinic.

## Materials and Methods

### Synthesis of C1

The synthetic methods used to make **C1**, or (*R*)-7-((4-(3-aminopyrrolidin-1-yl)-5-chloro-6-ethyl-7*H*-pyrrolo[2,3-*d*]pyrimidin-2-yl)thio)-1,5-naphthyridine are described elsewhere [15].

### Synthesis of C2, C3 and C4

The synthetic methods used to make **C2**, or (*S*)-1-(2-(pyrido[2,3-*b*]pyrazin-7-ylthio)-9*H*-pyrimido[4,5-*b*]indol-4-yl)pyrrolidin-3-amine, **C3**, or 6-fluoro-4-((3aR,6aR)-hexahydropyrrolo[3,4-*b*]pyrrol-5(1H)-yl)-*N*-methyl-2-((2-methylpyrimidin-5-yl)oxy)-9*H*-pyrimido[4,5-*b*]indol-8-amine, and **C4**, or 4-((1*S*,5*R*,6*R*)-6-amino-1-methyl-3-azabicyclo[3.2.0]heptan-3-yl)-6-fluoro-*N*-methyl-2-((2-methylpyrimidin-5-yl)oxy)-9*H*-pyrimido[4,5-*b*]indol-8-amine are described elsewhere [24].

### Generation, expression and purification of full length *E. faecalis* GyrB, *E. coli* GyrB, *F. tularensis* GyrB, *E. faecalis* ParE, *E. coli* ParE and *F. tularensis* ParE constructs for enzymological studies

Full length ORFs were PCR amplified and cloned into the NcoI and XhoI sites of pET28a (Novagen). The resulting clones contained a C-terminal hexahistadine tag. Sequence verified clones were transformed to the expression strain, *E. coli* BL21(DE3). Fermentation and purification conditions for all full-length proteins were as follows: Cells were grown at 37°C in 1 liter of Terrific Broth and induced with 1 mM IPTG once an OD_600_ of 0.8 was reached. The cells were harvested after an additional 12 hours of growth at 18°C. The cell pellets were resuspended in 50 mM Tris pH 8.0, 200 mM NaCl. Sonication was used to lyse the cells and the lysate was cleared by centrifugation. The supernatant was loaded onto a bed of Ni-NTA agarose and eluted with a gradient of imidazole in 50 mM Tris pH 8.0, 200 mM NaCl. The final chromatographic step was anion exchange (HiTrap Q HP from GE Healthcare) using a linear gradient of 25 mM Tris pH 8.0, 25-500 mM NaCl, while collecting fractions over 10 column volumes. The full-length GyrB and ParE proteins were soluble when overexpressed and were purified to > 98% purity (as determined by SDS-PAGE). 

### Generation, expression and purification of *E. faecalis* GyrB constructs, *E. coli* GyrB constructs and *F. tularensis* ParE constructs for crystallization studies

A uniform cloning method was employed for the generation of C-terminal hexahistadine tagged crystallization constructs. All PCR primers pairs were designed to introduce a BsaI recognition sequence, which when cut would produce compatible ends that could be ligated into pET28a treated with NcoI and XhoI. The primers used to generate the *F. tularensis* ParE construct were FtParE-fwd 5’- AATAATGGTCTCCCATGCAAAACTATAATGCTAAATCT-3’ and FtParE-rev 5’-AATAATGGTCTCCTCGAGTGCATTAATTCTTTTTTGTGC-3’, which encode amino acids 1-382 of the wild type protein. The *E. faecalis* GyrB construct was generated using the following primer pair: EfGyrB-fwd 5’-CAACACGGTCTCCCATGGGCTTAGAAGCTGTCCGGAAACGTC-3’ and EfGyrB-rev 5’-CCAACAGGTCTCCTCGAGCCGCCTTCATAGTGATACTCTTTTTTA-3’, resulting in a construct comprising residues 18-225 of the wild type sequence. The *E. coli* GyrB crystallization construct was amplified using the following primer pair:  EcGyrB-fwd 5’-AATAATGGTCTCCCATGG GGCTGGATGCGGTGCGTAA-3’

EcGyrB-rev 5’-AATAATGGTCTCCTCGAG GCCTTCATAGTGGAAGTGG-3’.  This clone contains residues 1-220 of the wild type protein. The expression and purification of this set of proteins was performed as described for the full-length proteins. *E. faecalis* GyrB and *E. coli* GyrB required further purification using a size-exclusion column (Superdex 75 from GE Healthcare) previously equilibrated with buffer consisting of 25 mM Tris pH 8.0, 100 mM NaCl.

### ATPase assay

GyrB and ParE activity were evaluated using the coupled spectrophotometric Enzchek™ assay in which the enzyme-dependent release of inorganic phosphate from ATP hydrolysis was measured. The assay contained between 20-100 nM GyrB or ParE (active site concentrations) in 50 µM Tris-HCl buffer (pH 7.6), 2 µM MgCl_2_, 125 µM NaCl, 0.2 µM 7-methyl-6-thioguanosine, 1U/mL purine nucleoside phosphorylase. The reaction was initiated by addition of 3 µM ATP and monitored at 360 nm for 30 min at 27°C. Inhibitor potency was determined by incubating the target enzyme in the presence of various concentrations of inhibitor ranging between 1.5 nM and 50 µM for 10 minutes prior to addition of ATP substrate. The final concentration of DMSO was kept constant at 2.5% (v/v). Enzyme activity in the presence of inhibitor was expressed relative to the no-inhibitor control and *K*_i_ values determined using Morrison tight-binding equation [25] to account for ligand depletion. All analysis was carried out using GraphPad Prism 4.0.

### Mechanism of action determination

The mechanism of action of GyrB/ParE targeting compounds was examined by monitoring macromolecular synthesis in *S. aureus* ATCC 29213 and a permeabilized *E. coli* strain, *E. coli* (*imp*) (BAS849). Macromolecular synthesis in the presence of increasing doses of compound was monitored by measuring the incorporation of radiolabeled precursors of DNA, RNA, protein and cell wall synthesis ([^3^H]-Thymidine, [^3^H]-Uridine, [^3^H]-Leucine, and [^3^H]-N-acetylglucosamine, respectively). A range of test compound, spanning the established minimal inhibitory concentration, was examined for each precursor. The extent by which synthesis of each pathway was inhibited was determined by calculating the difference in label incorporation relative to untreated controls. The effects were compared to of 5 control antibiotics specific for selected metabolic pathways: tetracycline (protein synthesis), ciprofloxacin and novobiocin (deoxyribonucleic acid [DNA] synthesis), rifampicin (ribonucleic acid [RNA] synthesis), and vancomycin (cell wall synthesis). A detailed description of the macromolecular synthesis assays used is provided elsewhere [26].

### Minimum inhibitory concentration assays

Assays for minimum inhibitory concentration (MIC) values reported in Tables 1 and 2 were performed as follows: Test compounds were solubilized in 100% dimethylsulfoxide (DMSO). Sterile deionized water was added to achieve a 50% DMSO stock solution and additional dilutions were made from the stock solutions to achieve a broader testing range. Mueller Hinton II broth (MHB) was used for testing with the exception of *H. influenzae*, which was tested in Haemophilus Test Medium Broth (HTM), and *S. pneumoniae*, which was tested in MHB supplemented with 2.5% lysed horse blood (LHB). Assay plates were loaded with 85 µL of test media appropriate for the test organism, 5 µL of drug solution, and 10 µL of bacterial inoculum. For TriBE inhibitors, the final concentration of DMSO was 2.5% with the exception of *H. influenzae* where the final concentration of DMSO was 0.6%. Final cell concentration in assay plates was approximately 5 x 10^5^ CFU/mL. Plates were incubated at 35°C for 18 to 24 hrs. and microplates were viewed from the bottom using a plate viewer. The MIC was read and recorded as the lowest concentration of drug that inhibited visible growth of the organism. Assays for MIC values reported in Table 3 were performed as follows: Test compounds were solubilized in 100% DMSO. Two-fold dilutions were prepared in 100% DMSO at 50X final MIC assay concentrations (final DMSO concentration of 2% v/v). MHB was used for testing with the exception of *H. influenzae*, which was tested in HTM, and *S. pneumoniae*, which was tested in MHB with 3% LHB. A subset of strains was also tested in the presence of 20% heat inactivated mouse serum. Assay plates were loaded with 98 µL of inoculated test media appropriate for the test organism (final cell concentration of approximately 5 x 10^5^ CFU/mL) and 2 µL of drug solution. Plates were incubated at 35°C for 18 to 24 hr and alamarBlue® was used to visualize cell viability. The MIC was read and recorded as the lowest concentration of drug that inhibited visible growth of the organism and color change of alamarBlue®. 

### Spontaneous incidence of resistance

Spontaneous mutations frequencies were assessed in *E. coli* (ATCC 25922). Large format assay dishes (245 x 245 mm) were prepared with 200 mL of Mueller Hinton II cation-adjusted agar containing **C3**, **C4** or ciprofloxacin (CIP) at a concentration 4X the MIC value. A suspension of ~1x10^10^ CFU in PBS was spread on each plate with glass beads. Plates were incubated at 37°C for 48 h. Putative mutant colonies were cultured on media containing an equivalent concentration of drug and run in MIC assays to confirm resistance phenotypes. Mutation frequencies were determined by dividing the number of resistant colonies by the actual CFU plated. Frequencies listed in Table 5 represent the average of values obtained for 3 plates, each derived from an independent culture. 

**Table 5:** Crystallographic statistics.

**Protein:Ligand complex**	***E. coli* GyrB C3**	***E. faecalis* GyrB C3**	***F. tularensis* ParE C3**	***E. faecalis* GyrB C1**	***E. faecalis* GyrB C2**	***E. faecalis* GyrB C4**
**PDB code**	4KFG	4K4O	4KQV	4KSH	4KTN	4KSG
**Data Collection** (data for high resolution shell in parentheses)						
Space group	P2_1_	P2_1_ 2_1_ 2_1_	P2_1_	P2_1_ 2_1_ 2_1_	P2_1_ 2_1_ 2_1_	P2_1_ 2_1_ 2_1_
Cell dimentions						
*a,b,c* (Å)	47.9, 82.5, 53.7	54.5, 58.4, 65.6	44.2, 163.2, 72.3	54.8, 58.3, 65.9	54.8, 58.9, 65.9	54.7, 58.7, 65.9
α, β, γ (°)	90, 100.16, 90	90, 90, 90	90, 91.8, 90	90, 90, 90	90, 90, 90	90, 90, 90
Resolution (Å)	82.48-1.6 (1.642-1.6)	43.58-1.30 (1.33-1.3)	81.58-2.38(2.45-2.38)	65.88-1.70(1.79-1.70)	43.94-1.69(1.77-1.68)	34.21-1.75(1.84-1.75)
R_sym_ or R_merge_ (%)	7.8(35.4)	5.6(50.9)	7.6(24.1)	7.5(63.2)	5.1(33.6)	3.4(6.0)
*I/σ(I)*	9.0(2.5)	18.5(2.9)	10.2(4.0)	12.6(2.3)	18.5(3.2)	23.0(12.3)
Completeness (%)	100(99.7)	99.2(97.6)	96(96)	98.6(91)	97.2(82.8)	100(100)
Redundancy	3.49(3.45)	3.7(3.1)	3.5(3.4)	3.7(3.5)	3.6(3.2)	3.5(3.4)
**Refinement**						
Resolution (Å)	44.5-1.6	43.58-1.30	81.58-2.38	65.88-1.70	43.94-1.69	43.84-1.75
No. reflections	51194	46571	37983	22529	22978	20881
R_work_/R_free_	19.2/22.1	17.8/19.6	25.5/29.8	17.1/20.5	17.4/20.3	17.6/20.9
**No. Atoms**						
Protein	2945	1503	4377	1524	1524	1503
Ligand(s)	79	37	64	32	32	38
Water	348	228	117	222	258	152
**B-factors**						
Protein	20.32	10.91	59	18.05	23.17	14.71
Ion	22.76 (SO4)	9.934 (TBU)	110.91	14.03	n/a	13.63 (TBU)
Inhibitor	19.34	9.54	43.35	13.33	32.47	13.15
Water	29.34	23.77	50.22	21.72	38.41	22.67
**R. m. s. deviations**						
Bond length (Å)	0.008	0.006	0.008	0.008	0.007	0.007
Bond angles (°)	1.559	1.417	1.373	1.321	1.301	1.66
Ramachandran plot (%)*						
favoured region	362 (98.4%)	182 (97.8%)	515 (95.5%)	182 (97.8%)	185 (97.9%)	182 (97.8%)
allowed region	6 (1.6%)	4 (2.2%)	22 (4.1%)	4 (2.2%)	4 (2.1%)	4 (2.2%)
outlier region	0 (0.0%)	0 (0.0%)	2 (0.4%)	0 (0.0%)	0 (0.0%)	0 (0.0%)

^*^ S.C. Lovell, I.W. Davis, W.B. Arendall III, P.I.W. de Bakker, J.M. Word, M.G. Prisant, J.S. Richardson & D.C. Richardson (2002)

### Mouse efficacy studies

Female CD-1 mice weighing 18 to 20 grams were pre-treated with cyclophosphamide to render the mice neutropenic. Mice were infected with *E. coli* (ATCC 25922) *via* injection into the right thigh muscle of 0.1 mL per mouse. One and a half hours post infection mice were treated IV with **C3** or **C4** at doses ranging from 1 to 15 mg/kg. Levofloxacin was used as a control, dosed once at 10 mg/kg. Four mice were treated with each drug concentration. Twenty-four hours post treatment, mice were euthanized by CO_2_ inhalation. The right thighs of the mice were aseptically removed, weighed, homogenized, serially diluted, and plated on TSA medium. The plates were incubated overnight at 37°C in 5% CO_2_. CFU per gram of thigh was calculated by enumerating the plated colonies then adjusting for serial dilutions and the weight of the thigh. 

### Crystallization and structure determination of *E. faecalis* GyrB C1, *E. faecalis* GyrB C2, *E. faecalis* GyrB C3 and *E. faecalis* GyrB C4 complexes

All complex crystal structures were determined from the C-terminal 6xHis tagged N-terminal 24 kDa ATP-binding domain of *E. faecalis* GyrB. Complexes with inhibitors were crystallized by hanging drop vapor diffusion, from 10-20 mg/mL protein solutions (concentrated in in 20 mM Tris, pH 8.0, 100 mM NaCl) combined with the appropriate small molecule ligand from a 10 mg/mL DMSO stock solution to a final concentration of 1 mM. Crystals were grown from drops using 1:1 ratios of the concentrated protein-ligand complex solution with the following reservoir/mother liquor solution at 20°C: 25% (w/v) PEG 1500, 3% (v/v) t-butanol, 20% (v/v) glycerol, 20 mM citrate pH 5.6. Crystals were harvested in nylon loops and frozen in liquid nitrogen without any additional cryoprotectant. Data on crystals of the *E. faecalis* GyrB complexes with **C1**, **C2** and **C4** were collected on a Rigaku RU 200 rotating anode generator equipped with a MAR345 image plate detector. Data were processed with Mosflm [27] and merged with programs from the CCP4 suite [28]. Data on crystals of the *E. faecalis* GyrB complex with **C3** were collected on the MBC 4.2.2 beamline at the Advanced Light Source, Lawrence Berkeley National Laboratory using a wavelength of 1 Å, a sample to detector distance of 120 mm and an oscillation angle of 0.5°. A complete data set was recorded on a NOIR-1 CCD detector. Diffraction data were processed, scaled and merged using D*TREK [29]. All the structures were determined by molecular replacement using *Phaser* [30] in the CCP4 program suite [28] with the coordinates of *E. faecalis* GyrB, PDB code 4GEE as a model [15]. Refinement was carried out using *REFMAC5.5* [31]. A total of 5% of the data were kept aside for *R*_free_ calculations. After initial rounds of restrained refinement, clear electron density was observed in the active site for inhibitors and ordered solvent, as well as t-butanol molecule. The model building, incorporation of solvent and ligands were performed with the program Coot [32]. The crystallographic asymmetric unit contains a single protein-ligand complex. Due to dynamic disorder, no electron density was observed for the last 5 histidine residues of the C-terminal histidine tag and residues between 101 (His) and 119 (Gly) belonging to a mobile surface loop. These residues were not included in the final models. The final refinement and geometry statistics are provided in Table 5. All models were validated using PROCHECK [33] and RAMPAGE [34] prior to submission to the Protein Data Bank.

### Crystallization and structure determination of the *E. coli* GyrB C3 complex

The *E. coli* GyrB **C3** complex crystal structure was determined from the C-terminal 6xHis tagged N-terminal 24 kDa ATP-binding domain of *E. coli* GyrB. The complex was crystallized by sitting drop vapor diffusion, from a 15 mg/mL protein solution (concentrated in in 20 mM Tris, pH 8.0, 100 mM NaCl) combined with **C3** from a 10 mg/mL DMSO stock solution to a final concentration of 1 mM. Crystals were grown from drops using 1:1 ratios of the concentrated protein-ligand complex solution with the following reservoir/mother liquor solution at 4°C: 15% (w/v) PEG 3350, 200 mM AmSO_4_, 100 mM Tris pH 7.5. Crystals were cryoprotected with mother liquor solution supplemented with 25% (v/v) ethylene glycol and harvested in nylon loops and frozen in liquid nitrogen. Data on a single crystal of *E. coli* GyrB complexed with **C3** were collected on the MBC 4.2.2 beamline at the Advanced Light Source, Lawrence Berkeley National Laboratory using a wavelength of 1 Å, a sample to detector distance of 120 mm and an oscillation angle of 0.5°. A complete data set was recorded on a NOIR-1 CCD detector. Diffraction data were processed, scaled and merged using D*TREK [29]. All the structures were determined by molecular replacement using *Phaser* [30] in the CCP4 program suite [28] with the coordinates of *E. coli* GyrB, PDB code 4KZN as a model [35]. Refinement was carried out using *REFMAC5.5* [31]. A total of 5% of the data were kept aside for *R*_free_ calculations. After initial rounds of restrained refinement, clear electron density was observed in the active site for inhibitors and ordered solvent. The model building, incorporation of solvent and ligands were performed with the program Coot [32]. The crystallographic asymmetric unit contains two protein-ligand complexes. Due to dynamic disorder, no electron density was observed for the last 5 histidine residues of the C-terminal histidine tag and residues between 94 (Leu) and 110 (Lys) belonging to a mobile surface loop. These residues were not included in the final models. The final refinement and geometry statistics are provided in Table 5. The final model was validated using PROCHECK [33] and RAMPAGE [34] prior to submission to the Protein Data Bank.

### Crystallization and structure determination of the *F. tularensis* ParE C3 complex

The *F. tularensis* ParE **C3** complex crystal structure was determined from the C-terminal 6xHis tagged N-terminal 39 kDa ATP-binding domain of *F. tularensis* ParE. The complex was crystallized by sitting drop vapor diffusion, from a 20 mg/mL protein solution (concentrated in in 20 mM Tris, pH 8.0, 100 mM NaCl) combined with **C3** from a 10 mg/mL DMSO stock solution to a final concentration of 1 mM. Crystals were grown from drops using 1:1 ratios of the concentrated protein-ligand complex solution with the following reservoir/mother liquor solution at 4°C: 10% (w/v) PEG 4000, 10% (v/v) isopropanol, 100 mM citrate pH 5.4. Crystals were cryoprotected with mother liquor solution supplemented with 30% (v/v) glycerol and harvested in nylon loops and frozen in liquid nitrogen. Data on a single crystal of *F. tularensis* ParE complexed with **C3** were collected on the MBC 4.2.2 beamline at the Advanced Light Source, Lawrence Berkeley National Laboratory using a wavelength of 1 Å, a sample to detector distance of 120 mm and an oscillation angle of 0.5°. A complete data set was recorded on a NOIR-1 CCD detector. Diffraction data were processed, scaled and merged using D*TREK [29]. All the structures were determined by molecular replacement using *Phaser* [30] in the CCP4 program suite [28] with the coordinates of *F. tularensis* ParE, PDB code 4HY1 as a model [15]. Refinement was carried out using *REFMAC5.5* [31]. A total of 5% of the data were kept aside for *R*_free_ calculations. After initial rounds of restrained refinement, clear electron density was observed in the active site for inhibitors and ordered solvent. The model building, incorporation of solvent and ligands were performed with the program Coot [32]. The crystallographic asymmetric unit contains two protein-ligand complexes. Due to dynamic disorder, no electron density was observed for residues 1-7, 103-111, 227-231, 258-260, 299-302, and 381 and the C-terminal histidine tag of the A chain. The B chain contains similar disorder, in addition to residues 218-381. Disordered residues were not included in the final model. The final refinement and geometry statistics are provided in Table 5. The final model was validated using PROCHECK [33] and RAMPAGE [34] prior to submission to the Protein Data Bank.

### Computational design methods

Computational work in support of this project benefited from supercomputing resources at Lawrence Livermore National Laboratory amounting to ~ 135K cpu-hours/week. Virtual high throughput screening using molecular docking and MM-GB/SA [36] rescoring were performed using Schrodinger’s Glide [37] and Prime [38] programs, and Amber10 [39] and Autodock [40]. Molecular dynamics calculations and thermodynamic integrations were performed during the optimization phase using Amber10 [39]. *Ab initio* ligand strain, polarizability and chemical stability calculations were performed using Gaussian03 at the B3LYP/6-31++G** level of theory). The QSAR and cheminformatics were performed using the Accelrys [41] suite of programs. 

### Ethics Statement

 The mouse efficacy studies were conducted under IACUC protocol # VVS11-008, which was reviewed and approved by Vivisource IACUC. All animal work was conducted at Vivisource which is a PHS-assured facility (PHS/OLAW assurance # A4543-01). All work complied with the animal care and use guidelines from *Guide for the Care and Use of Laboratory Animals* and that they comply with the applicable regulations (9 CFR, Subchapter A) issued by the U.S. Department of Agriculture (USDA) under the Animal Welfare Act.

## Supporting Information

Figure S1 **The locations of sequence diversity in GyrB and ParE enzymes from important clinical pathogens mapped on to the ATP-binding pocket of *E. coli* GyrB.** (Top) Semi-transparent view of the ATP-binding pocket from the crystal structure of the *E*. *coli* GyrB complexed with **C3**. **C3** is shown in gold. The positions of the side-chains (in the *E*. *coli* structure) of the six pocket-lining residues that are variable between the listed GyrB and ParE orthologs are shown in blue. All other pocket-lining residues are conserved in the species listed. (Bottom) Table showing the identities of the enumerated amino acid residues (from the top panel) for GyrB and ParE enzymes from all the listed bacterial species. The diversity observed at positions 1 and 3 had the greatest impact on ligand design. The Ile to Met change observed between GyrB and ParE at position 1 generates significant structural diversity in the “pocket floor” in the vicinity of the variable residue. Inhibitors with groups that impinge on the pocket floor in the vicinity of residue 1 generally demonstrated inferior dual-targeting activity. Diversity in residue 3 influences the volume of the interior lipophilic pocket: ParE enzymes from Gram-positive bacteria typically present a small Ala side-chain at this position, while the Gram-negative ParE enzymes present a large Ile side-chain at position 3. GyrB enzymes present intermediate Val or Ser residues at position 3. As a result, the Gram-negative ParE enzymes are the most spatially constrained in the vicinity of residue 3, and limit the size of substituents that are tolerated off the R_6_ position of the pyrimidoindole inhibitor scaffold.
(DOCX)
