# Supplementary figures and images for: Tricyclic GyrB/ParE (TriBE) Inhibitors: A New Class of Broad-Spectrum Dual-Targeting Antibacterial Agents
Source: PLoS One. 2013 Dec 26;8(12):e84409. doi: 10.1371/journal.pone.0084409 (PMC3873466; doi:10.1371/journal.pone.0084409)

Supplemental Figure 1


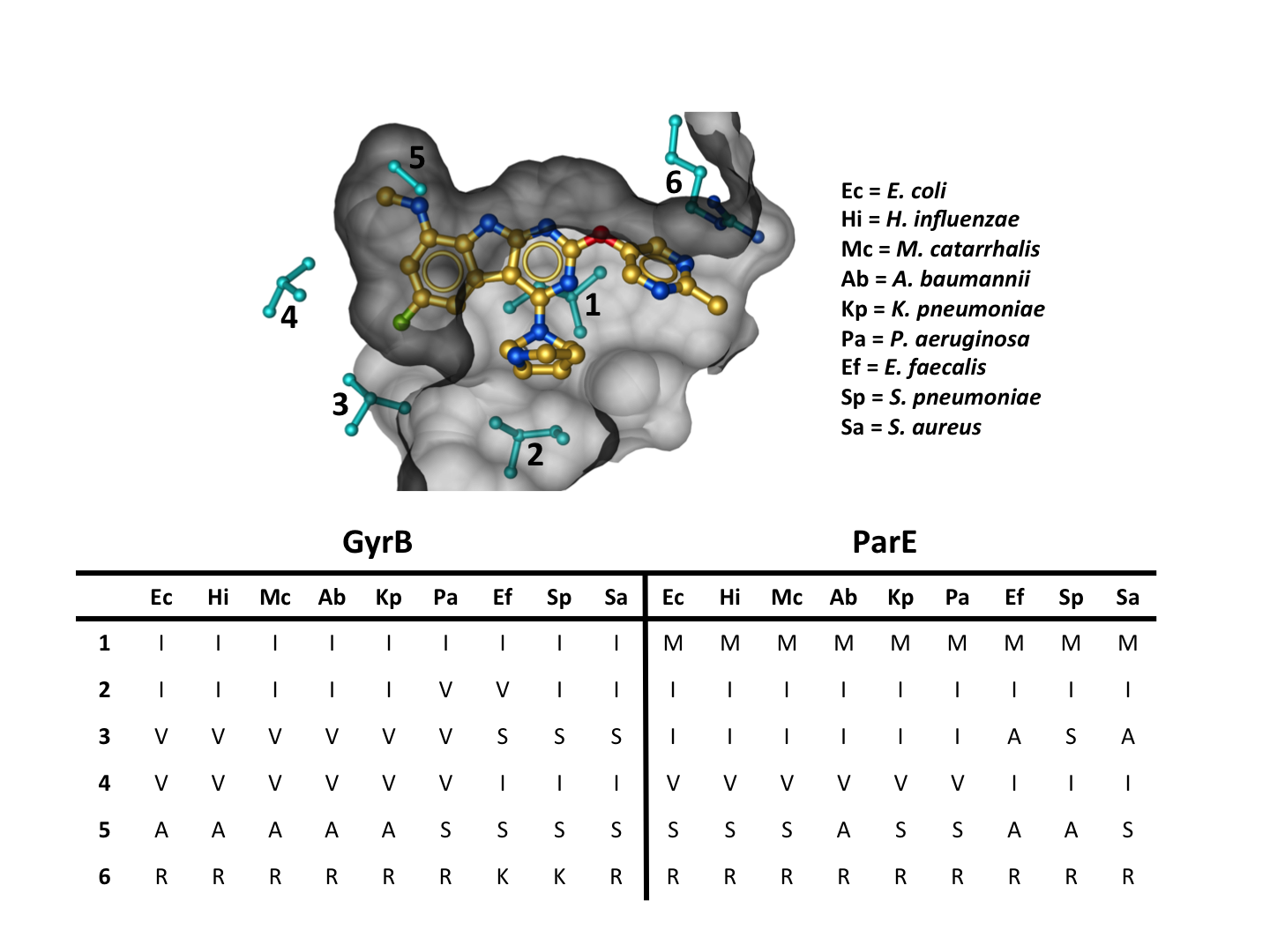

Supplement: Figure S1 — The locations of sequence diversity in GyrB and ParE enzymes from important clinical pathogens mapped on to the ATP-binding pocket of E. coli GyrB. (Top) Semi-transparent view of the ATP-binding pocket from the crystal structure of the E. coli GyrB complexed with C3. C3 is shown in gold. The positions of the side-chains (in the E. coli structure) of the six pocket-lining residues that are variable between the listed GyrB and ParE orthologs are shown in blue. All other pocket-lining residues are conserved in the species listed. (Bottom) Table showing the identities of the enumerated amino acid residues (from the top panel) for GyrB and ParE enzymes from all the listed bacterial species. The diversity observed at positions 1 and 3 had the greatest impact on ligand design. The Ile to Met change observed between GyrB and ParE at position 1 generates significant structural diversity in the “pocket floor” in the vicinity of the variable residue. Inhibitors with groups that impinge on the pocket floor in the vicinity of residue 1 generally demonstrated inferior dual-targeting activity. Diversity in residue 3 influences the volume of the interior lipophilic pocket: ParE enzymes from Gram-positive bacteria typically present a small Ala side-chain at this position, while the Gram-negative ParE enzymes present a large Ile side-chain at position 3. GyrB enzymes present intermediate Val or Ser residues at position 3. As a result, the Gram-negative ParE enzymes are the most spatially constrained in the vicinity of residue 3, and limit the size of substituents that are tolerated off the R6 position of the pyrimidoindole inhibitor scaffold. (DOCX) [file pone.0084409.s001.docx]
